# Acute Biliary Pancreatitis: Diagnosis and Treatment

**DOI:** 10.4103/1319-3767.54740

**Published:** 2009-07

**Authors:** Zakaria M. Hazem

**Affiliations:** Department of Surgery, College of Medicine, King Faisal University, Dammam, Kingdom of Saudi Arabia

**Keywords:** Acute biliary pancreatitis, diagnosis, treatment, predictors, severity

## Abstract

Gallstones are the commonest cause of acute pancreatitis (AP), a potentially life-threatening condition, worldwide. The pathogenesis of acute pancreatitis has not been fully understood. Laboratory and radiological investigations are critical for diagnosis as well prognosis prediction. Scoring systems based on radiological findings and serologic inflammatory markers have been proposed as better predictors of disease severity. Early endoscopic retrograde cholangiopancreatography (ERCP) is beneficial in a group of patients with gallstone pancreatitis. Laparoscopic cholecystectomy with preoperative endoscopic common bile duct clearance is recommended as a treatment of choice for acute biliary pancreatitis. The timing of cholecystectomy, following ERCP, for biliary pancreatitis can vary markedly depending on the severity of pancreatitis

Gallstones are the leading cause of pancreatitis worldwide, accounting for at least one half of the 4.8-24.2 cases of pancreatitis per 100,000 people that occur in Western countries.[1–4] About 80,000 cases occur in the USA; 17 per 100,000 new cases.[5] In Japan, the annual incidence lies between 5 - 80 per 100,000 of the population.[6,7]

Although majority of the patients with gallstone (biliary) pancreatitis recover without significant sequelae,15-30% have severe episodes requiring multidisciplinary care to ensure the best outcome.[8] Complications of acute biliary pancreatitis, both local (necrosis, pseudocyst formation, abscesses, hemorrhage) and systemic (pleural effusion, adult respiratory distress syndrome (ARDS), renal insufficiency, multiorgan failure) often require intensive care unit (ICU) management.[8,9]

Gender and stone size may be risk factors for gallstone pancreatitis. The risk of developing acute pancreatitis in patients with gallstones is greater in men; however, more women develop this disorder since gallstones occur with increased frequency in women.[10]

Acute pancreatitis is a potentially fatal disease with an overall mortality of 2 – 7% despite aggressive intervention.[11–14] The outcome of acute pancreatitis is determined by two factors which reflect the severity of the illness: organ failure and pancreatic necrosis. About half of the deaths in patients with acute pancreatitis occur within the first one/two weeks and are mainly attributable to multiple organ dysfunction syndromes. When not treated, the risk of recurrence in gallstone pancreatitis ranges from 32 to 61%.[10,12,13]

## ETIOLOGY OF ACUTE PANCREATITIS

The pathogenesis of acute pancreatitis has not been not fully understood. The mechanism by which the passage of gallstones induces pancreatitis is unknown. Suggested possible initiating events in gallstone pancreatitis include the reflux of bile into the pancreatic duct due to transient obstruction of the ampulla during passage of gallstones. In the past two decades there has been much interest about early removal of retained CBD stones and how beneficial it would be for patients with acute biliary pancreatitis. Many years later it was found that over 85% of patients with so called gallstone pancreatitis spontaneously passed stones that were recoverable in the stool.[14]

This discovery supported the ‘common channel theory’. Subsequently, it was demonstrated that sterile bile does not result in pancreatitis. However, infected bile is capable of activating pancreatic enzymes leading to auto digestion of the gland.[15] These two concepts: (a) reflux of infected bile into the pancreas activating a cascade of proteolytic enzymes, and (b) obstruction of pancreatic duct causing acinar disruption from raised pressure, are the favored explanations for the triggering of gallstone pancreatitis. A potentially unifying, yet unproven, hypothesis advanced by Moody in 1993 states that gallstones initiate pancreatitis through obstruction of the pancreatic duct and that progression to necrosis and severe pancreatitis requires the reflux of bile.[16,17]

In fact experimental models show a coalescence of zymogen granules with lysosome vacuoles resulting in intrapancreatic activation of proteolytic enzymes. Small amounts of trypsin can be countered by endogenous pancreatic trypsin inhibitor. However, large amounts of trypsin release can overwhelm the serological defense mechanism (α-1-antitrypsin and α-2-macroglobulin) and activate other enzymes resulting in local and systemic complications commonly seen in the course of the disease. Activation of the enzyme phospolipase A2 has important consequences like destruction of pulmonary surfactant that can result in ARDS and liberation of prostaglandins and leucotriens that may be important in the pathogenesis of the systemic inflammatory response which can lead to multi organ failure. More than that, inflammatory mediators may be used as predictors of disease severity in the near future. Also, trypsin activates and complements kinin, kallikrein, possibly playing a part in disseminated intravascular coagulation, shock, renal failure and vascular instability.[18,19]

## DISTINGUISHING BILIARY FROM OTHER FORMS OF ACUTE PANCREATITIS

The diagnosis of gallstone pancreatitis should be suspected if the patient has a prior history of biliary colic.[20,21] Although gallstone pancreatitis is the most common cause of pancreatitis, other etiologies must be considered, prior to initiating treatment, like moderate to heavy alcohol consumption over a period of years. Other causes include medication, genetic diseases, infectious agents, postoperative states, endoscopic procedure involving pancreatic and bile ducts and other types of injury to pancreas.[22,23] It goes without saying that a detailed history and careful physical examination are the first step towards making the diagnosis.[24,25] Laboratory and radiological investigations are critical for diagnosis as well as prediction of prognosis when a patient presents with gallstone pancreatitis. Documenting an elevated serum amylase and/or lipase is helpful in diagnosing pancreatitis. Serum amylase is elevated in at least 75% of cases of acute pancreatitis and remains elevated for 5-10 days in most patients. However, amylase lacks specificity for pancreatitis because it can be elevated in other disorders. Lipase is more specific for pancreatitis, but both enzymes may be increased in renal failure and various abdominal conditions (e.g., perforated ulcer, mesenteric vascular occlusion and intestinal obstruction). Other causes of increased serum amylase include salivary gland dysfunction, macro amylasemia, and tumors that secrete amylase.[26,27] Serum lipase has a longer half life than amylase and therefore tends to remain elevated for longer. Using a cut-off of three times the upper limit of normal, the sensitivity of serum lipase for pancreatitis approaches 90% in patients presenting with abdominal pain.[28] A urine dipstick for trypsinogen-2 has sensitivity and specificity of more than 90% for acute pancreatitis.[28,29]

Several tests can help differentiate biliary pancreatitis from other causes of pancreatitis. Aspartate aminotransferase (AST), alanine aminotransferase (ALT), alkaline phosphatase and serum bilirubin are the so-called liver function tests; they should be reviewed before making a confident diagnosis.

In a recent study, the specificity of a serum ALT level of more than 150 IU/L for diagnosing gallstone pancreatitis was 96%.[29] Unfortunately, the sensitivity was only 48%. This means that if the patient has a serum high ALT in the appropriate setting one can be fairly sure that the etiology of pancreatitis is biliary, but a normal ALT does not exclude gallstones as a cause.[17,18,23] Experimental biochemical markers that may hold promise for assessing the severity of disease include trypsinogen activation peptide, interleukin-6, interleukin-10, procalcitonin, phospholipase A_2_ and C-reactive protein levels.[30,31] Currently, these markers have limited clinical availability, but there is significant interest in better understanding markers of immune response and pancreatic injury because these could be valuable tools for reliably predicting the severity of acute pancreatitis and supplementing imaging modalities.[31–33]

The finding of gallstones and dilatation of the extra hepatic biliary tree on cross-sectional abdominal imaging lends further support to the diagnosis of gallstone pancreatitis. However, the sensitivity for detection of dilated bile ducts from biliary obstruction ranges in various studies from 55 to 91% [Table 1]. Trans abdominal ultrasonography seldom visualizes the pancreas in patients with acute pancreatitis due to air in the distended loops of the small bowel [Table 1].[34]

**Table 1:** Comparison of imaging techniques for acute pancreatitis[50–52,55–57]

Imaging technique	Effectiveness
Contrast-enhanced computed tomography	78 per cent sensitivity and 86 per cent specificity for severe acute pancreatitis
Endoscopic ultrasonography	100 percent sensitivity and 91 per cent specificity for gallstones
Magnetic resonance cholangiopancreatography	81 to 100 per cent sensitivity for detecting common bile duct stones 98 per cent negative predictive value and 94 per cent positive predictive value for bile duct stones
	As accurate as contrast-enhanced computed tomography in predicting severity of pancreatitis and identifying pancreatic necrosis
Magnetic resonance imaging	83 per cent sensitivity and 91 per cent specificity for severe acute pancreatitis
Transabdominal ultrasonography	87 to 98 per cent sensitivity for the detection of gallstones

Helical computerized tomography (CT) is one choice for accurate imaging diagnosis and staging of pancreatitis. A CT allows identification of pancreatic edema, fluid or cysts, and the severity of pancreatitis to be graded, detects complications including development of pseudocysts, abscess, necrosis, hemorrhage, and vascular occlusion.[35]

The CT criteria for diagnosis of pancreatic necrosis are dependent on the detection of areas which lack glandular enhancement, which may be focal or more diffuse. Balthazar *et al*.[36] showed that there was close correlation between the presence and extent of necrosis and course of hospitalization including morbidity and mortality. Based on this work a CT severity index was developed which attempted to use CT as a numeric grading system for the radiological grading of pancreatitis. The system provides a score between 0 and 10 with higher morbidity and mortality found with higher scores. A severity score of 7-10 had 92% complication rate and 17% mortality rate while a score of 0-1 had nil morbidity or mortality.

Caution in defining pancreatic necrosis is important as areas of peri pancreatic fluid can simulate areas of necrosis. Pancreatic necrosis is ideally detected on scans performed 48-72 hours after the onset of an attack of acute pancreatitis. Scans done within the first 24 hours may be falsely negative or equivocal. Although scans are commonly done at time of admission, the need for a second study should be kept in mind for patients without rapid improvement initially.[37,38]

The CT finding of CBD stones may have sensitivity as high as 80%, as noted by Baron *et al,*.[35] who reviewed 69 patients with biliary obstruction, 12 of whom subsequently proved to have CBD stones (10 identified by the CT scan). However, according to experiences of many gastroenterologists, CT is often less sensitive than trans abdominal ultrasound. Contrast-enhanced CT scans are more valuable than noncontrasted ones for assessing the severity of acute pancreatitis. CT scans can be normal in 15-20% of patients with mild pancreatitis. Not all patients with acute pancreatitis require a CT scan. This can be reserved when the diagnosis is in doubt, severe pancreatitis is suspected or conservative management fails.[39]

In a retrospective analysis of 76 patients undergoing retrograde cholangiopancreatography, Neoptolemos *et al.,* devised a scoring system to predict the presence of CBD stones based on bilirubin less than 40 μmoI/L (2.5 mg/dL), gamma-glutamyl trans-peptidase (I -GT) less than 250 IU/I, alkaline phosphatase less than 225IU/L and age less than 70years.[40] The presence of all four had a positive predictive value of 93%; the prediction of choledocholithiasis by a bilirubin less than 40 μmoI/L in patients with severe pancreatitis was 85%. Subsequent studies analyzed preoperative predictors of choledocholithiasis. Unfortunately, these studies were retrospective and not validated prospectively. Also, they contain statistical assumptions which make their clinical applicability questionable.

The most thorough analysis was probably that of Ohken *et al.*[41] They devised a predictive model after reviewing records of 465 patients, 115 (25%) of whom had confirmed CBD stones. The final model used CBD diameter, maximum serum bilirubin level, AST and alkaline phosphatase to predict choledocholithiasis with a success rate of 76%.

## SCORING SYSTEMS

Several scoring systems can predict the severity of pancreatitis and recent work has attempted to compare their relative predictive values.

The greater the number of Ranson criteria present, the higher the predicted mortality. Patients with two or less Ranson criteria have around one per cent mortality; those with more than five criteria have a mortality of around 40%.[42]

Other grading systems, such as the Glasgow (or Imrie) score,[43] may be more accurate in the specific case of gallstone pancreatitis. Unfortunately both Ranson and Glasgow systems take 48 hours for full assessment.

The Acute Physiology and Chronic Health Evaluation (APACHE) system which is more cumbersome to use than Ranson or Glasgow scale has the advantage of rapid assessment of severity at the time of admission to the ICU (or other high dependency setting).[44–46]

Each of the three systems has its specific advantages and disadvantages, but they are roughly equivalent in accuracy for predicting the severity of acute pancreatitis.

Research has shown some advantages of the CT severity index in predicting the severity of acute pancreatitis compared with the other systems. One study found that a CT Severity Index score of five or greater correlated with prolonged hospitalization and higher rates of mortality and morbidity.[47–49] A CT severity index score of five or greater was associated with a mortality rate 15 times higher than in those with a score of less than five. No association was found between Ranson's criteria and APACHE II scale scores and mortality or length of hospitalization.[48,49]

MRCP, a truly noninvasive method, is the most attractive alternative to ERCP for diagnosing choledocholithiasis. MRCP has been found to be as accurate as contrast-enhanced CT in predicting the severity of pancreatitis and identifying pancreatic necrosis.[50] Unlike ERCP, MRCP does not have interventional capability for stone extraction, stent insertion, or biopsy. MRCP is less sensitive for detection of small stones (i.e., smaller than 4 mm), small ampullary lesions, and ductal strictures.[51] MRCP can assess pancreatic and peri pancreatic cysts.[52] It is helpful for patients when ERCP is not possible or is unsuccessful.[50]

The Agency for Healthcare Research and Quality (AHRQ) reviewed nine studies evaluating the performance of MRCP with ERCP as the reference standard. Seven of these studies showed high concordance between MCRP and ERCP with both sensitivity and specificity being greater than 90%. The conclusion was that while these studies demonstrated good concordance between the two tests, evidence does not allow one to conclude which test is better. Interestingly, Sugiyama *et al.*[53] analyzed the sensitivity of MRCP based on stone size, showing lower sensitivity for stone less than a centimeter. This is an important consideration as most stones that cause pancreatitis are likely to be small

Another new technology is endoscopic ultrasonography, which is highly accurate in documenting stones and tumors but is used less often than ERCP. Endoscopic ultrasonography is useful in obese patients and patients with ileus, and can help determine which patients with acute pancreatitis would benefit most from therapeutic ERCP.[54] Endoscopic ultrasonography can assist with endoscopic trans mural cyst and abscess drainage. Endoscopic ultrasonography and MRCP show promise in increasing the range of options available to search for the cause of acute pancreatitis.[50,55]

## SEROLOGY MARKERS

Recently, as the role of inflammatory response and oxidative stress in the pathogenesis of acute pancreatitis emerge; inflammatory markers have been proposed as better predictors of disease severity. The most promising of these are C reactive protein, interleukin-6, and, in urine, albumin, immunoglobulin, trypsinogen activation peptide.[58–63]

Polymorphonuclear leucocyte elastase levels are significantly higher in severe pancreatitis than in mild cases, but as this test is not widely available, it, too, lacks clinical utility.[61,62]

Manes *et al*.[64] assessed the value of serum interleukin-6 in comparison with C reactive protein in a prospective clinical study. This was to discriminate necrotizing from edematous, acute pancreatitis due to common bile duct stones, in the first hours of disease. C reactive protein showed low efficacy in detecting necrotizing forms. The study concluded that serum interleukin-6 is a very reliable marker of necrosis in the first 48 hours of acute biliary pancreatitis.

## TREATMENT OF ACUTE PANCREATITIS

The treatment of gallstone pancreatitis is usually conservative, including bowel rest and intravenous fluid replacement. Fluid resuscitation is vital but often inadequate or over looked.[65,66]

A British group was the first to prospectively evaluate the role of ERCP in acute biliary pancreatitis.[65] About 121 patients with acute pancreatitis and ultrasound evidence of gallstone disease were randomized to either conventional medical management or urgent ERCP within 72 hours. Patients were stratified by severity of illness; one-half of the patients randomized to ERCP had severe pancreatitis. CBD stones were found in 63% of patients with severe pancreatitis, but only 25% of those with mild pancreatitis. Sphincterotomy was performed in patients with bile duct stones. In the group randomized to intervention with ERCP and sphincterotomy, there was a significant reduction in complications in those with severe disease, 24% with 4% mortality vs. 61% with 18% mortality. However, there was no difference in outcome of patients with mild pancreatitis.

This meant that many patients were being subjected to an invasive procedure after they passed the offending bile duct stone.

In an effort to increase the yield of ERCP in this setting, a variety of predictive scoring systems have been developed. Although critics suggest that the improvement seen in urgent ERCP patients was solely because of relief by cholangitis, when this diagnosis is excluded, a statistically significant benefit can still be shown for patients with predicted severe acute pancreatitis.

The second single center randomized controlled trial of endoscopic therapy in gaIlstone pancreatitis was published in 1993, by Fan *et al*.[67] In an effort to determine whether early (i.e., within 24 hours of being admitted) ERCP and biliary stone extraction would improve outcome in gallstone pancreatitis when compared to a conservative approach, 195 patients were randomized between the two study arms.

The conservative treatment patients underwent ERCP and endoscopic stent (ES) only if their clinical condition deteriorated. Complications were classified as local- (pancreatic abscess, pseudocyst, phlegmon, pseudo aneurysm), systemic (renal failure, respiratory failure, shock, coagulopathy) and biliary (sepsis requiring surgical or endoscopic intervention).

The prediction of the severity of the attack was based on plasma glucose and urea levels on admission, and on Ranson's multifactorial scoring system applied at 48hours. A total of 127 patients proved to have bile duct stones. Of 97 patients undergoing urgent ERCP, 37 (38%) were found to have stones impacted at the ampulla or in the common bile duct; all underwent successful papillotomy and stone extraction. ERCP failed in 10 patients, one of whom was subsequently determined to have a biliary stone. Five patients died, all of whom had predicted severe pancreatitis.

Of 98 patients randomized to conservative treatment, 27 (28%) (9 ‘severe’) required ERCP within the next 12 days (ERCP failed in two patients): Nine patients (9%) with predicted severe pancreatitis died; four had CBD stones. Complications occurred in 18% of those randomized to early ERCP and in 29% of those assigned to conservative management (*P* = 0.07). Biliary sepsis was more common in the conservative treatment group (12%) than in patients who underwent ERCP, with or without ES (0%). The authors concluded that emergency ERCP with or without ES is indicated in acute pancreatitis patients.

This study failed to demonstrate a statistically significant increase in survival or reduction in complications following early ERCP with or without ES, but did show a dramatic reduction in the risk of cholangitis. The study has been criticized because of the high prevalence of gallstones in Hong Kong, limiting the applicability of the resuIts to Western population. Scrutiny of data helps understand the severity of disease and presence or absence of cholangitis and abnormal liver tests.[68,69]

This study did confirm, however, that in expert hands, urgent ERCP for selected patients is safe and beneficial.

A third prospective, randomized controlled trial was performed in Poland and published in an abstract form in 1995.[79] This study involved 280 patients managed in the following ways: 75 were subjected to early endoscopic sphinterotomy because they had a stone impacted at the ampulla of Vater; the remaining 205 patients, who had a grossly normal appearing duodenal papilla, were randomized to immediate ES or conservative management. The patient groups were equivalent with respect to predicted severity of their pancreatitis, age and gender. Combining the data from all patients who had early ES, 17% had complications of pancreatitis, compared with 36% of those who were randomized to conservative therapy.

Mortality was significantly higher in patients randomized to the conservative approach (13% vs. 2%). In contrast to the previous two studies, the differences in the Polish study were the same for those with predicted mild and predicted severe pancreatitis. Even more impressive results were seen when the onset of gallstone pancreatitis and ES were separated by less than 24 hours (mortality 0%, complications 7%).

The authors concluded that early ES should be performed in all patients with gallstone pancreatitis (predicted mild and severe) since it decreased morbidity and mortality. Problems with this study include the fact that it has not been published in a peer-reviewed journal, a lack of true randomization, and the fact that some of the patients with empty ducts may have had more severe irreversible damage, or pancreatitis due to other etiologies than stone disease.

The results are consistent with previous studies in suggesting that early ERCP is beneficial in a group of patients with gallstone pancreatitis. The major difference between this study and its predecessors is that all the patients improved, whereas only select patient groups (e.g. those with biliary sepsis and those with predicted severe pancreatitis) seemed to benefit previously.

The most contentious study is the German multicenter study published by Folsch *et al.*,[70] in which 238 patients with suspected biliary pancreatitis were randomized to early ERCP within 72 hours of presentation or conservative management. Patients with jaundice were excluded 58 of 121 patients randomized to the ERCP arm were found to have bile duct stones. In the control arm, 13 of 112 were crossed over to ERCP for apparent bile duct stones. In this study, there was no improvement in outcome from early sphincterotomy. Paradoxically, there appeared to be more severe complications including respiratory failure in the early ERCP group, and a numerically increased mortality.

The authors conclude that early ERCP was not indicated in patients with gallstone pancreatitis who did not have evidence of biliary obstruction. This study has been criticized for several reasons. First, the authors excluded patients with obstructive jaundice (bilirubin> 5.0mg/dL) and those most likely to have CBD stones and benefit from biliary decompression. This biased the study towards the conservatively treated group. Secondly, whereas the prior studies were from a single centre with experienced endoscopists, the Folsch study took patients from 22 centers, some of which contributed as few as two patients per year. The low volume of urgent ERCPs in these centers raises concern about the experience and expertise of the endoscopists working there. Admittedly, this may represent the reality of ERCP practice in many community settings, where therapeutic cases are infrequent. Thirdly, the finding of a statistically significant increase in respiratory failure (inability to maintain arterial partial pressure of oxygen above 60 mmHg) in those undergoing early ERCP was not seen in any of the prior studies of ERCP in gallstone pancreatitis.

Data regarding the exact nature of the respiratory problems, relevant co morbidities and their relation to the predicted severity of pancreatitis were not offered in the published paper. On the other hand, it is feasible that the significant incidence of respiratory failure may be a statistical aberration.

Fourthly, as the Folsch study was terminated early it lacks the necessary statistical power to conclude that early ERCP with ES is not beneficial in this particular group of patients with gallstone pancreatitis.[71,72]

Unless there is reasonably clear evidence of a persistent bile duct stone such as a rising serum bilirubin or an imaging study clearly showing an intraductal stone, routine use of ERCP is unnecessary and adds avoidable risk in patients with mild to moderate biliary pancreatitis in whom cholecystectomy is planned. For majority of the patients with suspected biliary pancreatitis, bile duct stones have passed by the time cholangiography is performed. ERCP can be deferred and any remaining ductal stones can be identified at intraoperative cholangiography during laparoscopic cholecystectomy. These stones can then be removed by postoperative or even intraoperative ERCP, or in those few centers with the appropriate expertise, by laparoscopic common bile duct exploration. If ERCP is unsuccessful, the patient can be referred to a tertiary endoscopy center where biliary access is virtually always possible.[73–75]

UK guidelines for gallstone pancreatitis advocate definitive treatment during the index admission, or within two weeks of discharge. However, this target may not always be achievable.[76] The study by Sanjay *et al*.(2008) reviewed current management of gallstone pancreatitis in a university hospital and evaluated the risk associated with interval cholecystectomy. This study demonstrates that overall 62% of patients with gallstone pancreatitis have definitive therapy during the Index admission. However, surgery was deferred in the majority (n=30) of patients with severe gallstone pancreatitis, and 19/30 underwent ES prior to discharge. ES and interval cholecystectomy in severe gallstone pancreatitis is associated with minimal morbidity and readmission rates, and is considered a reasonable alternative to an index cholecystectomy in patients with severe gallstone pancreatitis.[77]

ERCP is appropriate in postcholecystectomy patients with suspected biliary pancreatitis, but in many of these patients the etiology is of a non biliary stone etiology such as sphincter of Oddi dysfunction, a setting in which conventional diagnostic and therapeutic ERCP techniques can be highly risky,[78–82] and protective measure such as placement of a pancreatic stent may be advisable.[76,83,84]

Recurrent biliary pancreatitis in patients with moderately severe gallstone pancreatitis is negligible after ERCP and ES. Hospital discharge of these patients permits interval laparoscopic cholecystectomy, but close follow-up is necessary in these potentially ill patients.[85]

Laparoscopic cholecystectomy with preoperative endoscopic CBD clearance is recommended as a treatment of choice for biliary acute pancreatitis. In mild disease, this is performed safely within seven days, whereas in severe disease, especially in extended pancreatic necrosis, at least three weeks should elapse because of an increased infection risk.[86]

According to Chang *et al.,*[87] patients with mild to moderate gallstone pancreatitis without cholangitis, selective postoperative ERCP and CBD stone extraction are associated with a shorter hospital stay, less cost, no increase in combined treatment failure rate, and significant reduction in ERCP use compared with routine preoperative ERCP. However, the study is limited by its small sample size.

The timing of cholecystectomy following ERCP for biliary pancreatitis can vary markedly depending on the severity of pancreatitis and overall health of the patient. Cholecystectomy follows only several weeks after the necrotizing pancreatitis has resolved. The risk of recurrent biliary pancreatitis should be quite low if ES is performed at the time of the ERCP. Most agree now that a laparoscopic cholecystectomy should be performed following an ES for gallstone pancreatitis if the patient is a reasonable operative risk.[88–90]

Patients with biliary pancreatitis that resolves rapidly should undoubtedly be treated with a cholecystectomy prior to dismissal from the index hospitalization.[91,92]

## CONCLUSIONS

Mild pancreatitis can usually be managed conservatively; a few of these patients require urgent ERCP. If there is concern regarding the possibility of a retained CBD stone, ERCP can be performed safely and almost always successfully following laparoscopic cholecystectomy. The timing of cholecystectomy following ERCP for biliary pancreatitis can vary markedly depending on the severity of pancreatitis. Patients with severe pancreatitis and those with ascending cholangitis are likely to benefit from early ERCP and ES to decompress the biliary tree. Cholecystectomy may follow only several weeks after the necrotizing pancreatitis has resolved. The risk of recurrent biliary pancreatitis should be quite low if ES is performed at the time of the ERCP.

It is increasingly appreciated that inflammatory mediators-such as interleukins, TNF-α, etc. -play a pivotal role in the development of clinical pancreatitis. Serum interleukin-6 is a very reliable marker of necrosis in the first 48 hours of acute biliary pancreatitis. In expert hands, the complication rate of ERCP, even in acutely ill patients, appears to be low.

The experience at large academic centers where expert endoscopists perform several procedures each year is unlikely to be mirrored in smaller community hospitals - all the more reason for community gastroenterologists to choose their urgent ERCPs with care.

MRCP has been found to be as accurate as contrast-enhanced CT in predicting the severity of pancreatitis and identifying pancreatic necrosis but is less sensitive for detection of small stones. Endoscopic ultrasonography is useful in obese patients and patients with ileus, and can help determine which patients with acute pancreatitis would benefit most from therapeutic ERCP.

## References

[1] Thomson SR, Hendry WS, McFarlane GA, Davidson AI (1987). Epidemiology and outcome of acute pancreatitis. Br J Surg.

[2] Opie EL (1901). The etiology of acute hemorrhagk pancreatitis. Bull. John Hopkins Hosp.

[3] Imrie C, Why A (1975). A prospective study of acute pancreatitis. Br J Surg.

[4] Moreau JA, Zinsmeister AR, Melton LJ, DiMagno EP (1988). Gallstone pancreatitis and the effect of cholecystectomy: a population-based study. Mayo Clin Proc.

[5] Eland IA, Sturkenboom MJ, Wilson JH, Stricker BH (2000). Incidence and mortality of acute pancreatitis between 1985 and 1995”. Scand J Gastroenterol.

[6] Sekimoto M, Takada T, Kawarada Y, Hirata K, Mayumi T, Yoshida M (2006). JPN Guidelines for the management of acute pancreatitis: epidemiology, etiology, natural history, and outcome predictors in acute pancreatitis. J Hepatobiliary Pancreat Surg.

[7] Imamura M (2004). Epidemiology of acute pancreatitis--incidence by etiology, relapse rate, cause of death and long-term prognosis. Nippon Rinsho.

[8] Death from acute pancreatitis (1977). MRC Multicenter Trial Glucagon Aprotinin. Lancet.

[9] McFadden DW (1991). Organ failure and multiple organ system failure in pancreatitis. Pancreas.

[10] Reila A, Zeinthmeister AR, Milton Lj (1991). Etiology, incidence and survival of acute pancreatitis in olmested county, Minnosota. Gastroentrology.

[11] de Beaux AC, Palmer KR, Carter DC (1995). Factors influencing morbidity and mortality in acute pancreatitis; an analysis of 279 cases. Gut.

[12] Agarwal N, Pitchumoni CS (1991). Assessment of severity in acute pancreatitis. Am J Gastroenterol.

[13] Gislason H, Horn A, Hoem D, Andrén-Sandberg A, Imsland AK, Søreide O (2004). Acute pancreatitis in Bergen, Norway. A study on incidence, etiology and severity. Scand J Surg.

[14] Funnell IC, Bornman PC, Weakley SP, Terblanche J, Marks IN (1993). Obesity: an important prognostic factor in acute pancreatitis. Br J Surg.

[15] Opie E (1901). The etiology of acute hemorrhagic pancreatitis. Johns Hopkins Hosp Bull.

[16] Sugawa C, Park DH, Lucas CE, Higuchi D, Ukawa K (2001). Endoscopic sphincterotomy for stenosis of the sphincter of Oddi. Surg Endosc.

[17] Takeda K, Matsuno S, Sunamura M, Kobari M (1998). Surgical aspects and management of acute necrotizing pancreatitis: Recent results of a cooperative national survey in Japan. Pancreas.

[18] Banerjee AK, Galloway SW, Kingsnorth AN (1994). Experimental models of acute pancreatitis. Br J Surg.

[19] Formela LJ, Galloway SW, Kingsnorth AN (1995). Inflamatory mediators in acute pancreatitis. Br J Surg.

[20] Acosta JM, Ledesma CL (1974). Gallstone migration as a cause of acute pancreatitis. N Engl J Med.

[21] Kelly TR (1980). Gallstone pancreatitis: the timing of surgery. Surgery.

[22] Acosta MJ, Rossi R, Ledesma CL (1977). The usefulness of stool screening for diagnosing cholelithiasis in acute pancreatitis. A Description Technique. Am J Dig Dis.

[23] Whitcomb DC (2006). Clinical practice. Acute pancreatitis. N Engl J Med.

[24] Acosta JM, Pellegrini CA, Skinner DB (1980). Etiology and pathogenesis of acute biliary pancreatitis. Surgery.

[25] Kingsnorth A, O'Reilly D (2006). Acute pancreatitis. BMJ.

[26] Moody FG, Senninger N, Runkel N (1993). Another challenge to the Opie's theory. Gastroenterology.

[27] Crunkel N, Moody F, Mueller W (1992). Experimental evidence against Opie's common channel bile reflux theory. Digestion.

[28] Sarles H (1991). Definitions and classifications of pancreatitis. Pancreas.

[29] Agarwal N, Pitchumoni CS, Sivaprasad AV (1990). Evaluating tests for acute pancreatitis. Am J Gastroenterol.

[30] Smotkin J, Tenner S (2002). Laboratory diagnostic tests in acute pancreatitis. J Clin Gastroenterol.

[31] Clavien PA, Burgan S, Moossa AR (1989). Serum enzymes and other laboratory tests in acute pancreatitis. Br J Surg.

[32] Neoptolemos JP, Kemppainen EA, Mayer JM, Fitzpatrick JM, Raraty MG, Slavin J (2000). Early prediction of severity in acute pancreatitis by urinary trypsinogen activation peptide: a multicentre study. Lancet.

[33] Tenner S (2004). Initial management of acute pancreatitis: critical issues during the first 72 hours. Am J Gastroenterol.

[34] Chak A, Hawes RH, Cooper GS, Hoffman B, Catalano MF, Wong RC (1999). Prospective assessment of the utility of EUS in the evaluation of gallstone pancreatitis. Gastrointest Endosc.

[35] Baron RL, Stanley RJ, Lee JK, Koehler RE, Levitt RG (1983). Computed tomographic features of biliary obstruction. AJR Am J Roentgenol.

[36] Balthazar EJ, Robinson DL, Megibow AJ, Ranson JH (1990). Acute pancreatitis: Value of CT in establishing prognosis. Radiology.

[37] Kim T, Murakami T, Takahashi S, Okada A, Hori M, Narumi Y (1999). Pancreatic CT imaging: Effects of different injection rates and doses of contrast material. Radiology.

[38] Balthazar EJ (2002). Acute Pancreatitis: Assessment of severity with clinical and CT evaluation. Radiology.

[39] Kemppainen E, Sainio V, Haapiainen R, Kivisaari L, Kivilaakso E, Puolakkainen P (1996). Early localization of necrosis by contrast-enhanced computed tomography can predict outcome in severe pancreatitis. Br J Surg.

[40] Neoptolemos JP, London N, Bailey I, Shaw D, Carr-Locke DL, Fossard DP (1986). The role of clinical and biochemical criteria and endoscopic retrograde cholal1giopancreatography in the urgent diagnosis of common bile duct stones in acute pancreatitis. Surgery.

[41] Onken JE, Brazer SR, Eisen GM, Williams DM, Bouras EP, DeLong ER (1996). Predicting the presence of choledocholithiasis in patients with symptomatic cholelithiasis. Am J Gastroenterol.

[42] Ranson JH (1982). Etiological and prognostic factors in human acute pancreatitis: a review. Am J Gastroenterol.

[43] Blamey SL, Imrie CW, O'Neill J, Gilmour WH, Carter DC (1984). Prognostic factors in acute pancreatitis. Gut.

[44] Knaus WA, Zimmerman JE, Wagner DP, Draper EA, Lawrence DE (1981). APACHE-acute physiology and chronic health evaluation: a physiologically based classification system. Crit Care Med.

[45] Larvin M, McMahon MJ (1989). APACHE-II score for assessment and monitoring of acute pancreatitis. Lancet.

[46] Wilson C, Heath DI, Imrie CW (1990). Prediction of outcome in acute pancreatitis: a comparative study of APACHE 11, clinical assessment and multiple factor scoring systems. Br J Surg.

[47] Leung TK, Lee CM, Lin SY, Chen HC, Wang HJ, Shen LK (2005). Balthazar computed tomography severity index is superior to Ranson criteria and APACHE II scoring system in predicting acute pancreatitis outcome. World J Gastroenterol.

[48] Chatzicostas C, Roussomoustakaki M, Vardas E, Romanos J, Kouroumalis EA (2003). Balthazar computed tomography severity index is superior to Ranson criteria and APACHE II and III scoring systems in predicting acute pancreatitis outcome. J Clin Gastroenterol.

[49] Vriens PW, van de Linde P, Slotema ET, Warmerdam PE, Breslau PJ (2005). Computed tomography severity index is an early prognostic tool for acute pancreatitis. J Am Coll Surg.

[50] Makary MA, Duncan MD, Harmon JW, Freeswick PD, Bender JS, Bohlman M (2005). The role of magnetic resonance cholangiography in the management of patients with gallstone pancreatitis. Ann Surg.

[51] Gosset J, Deviere J, Matos C (2004). Magnetic resonance imaging of acute pancreatitis: the pancreatogram. JOP.

[52] Barish MA, Yucel EK, Ferrucci JT (1999). Magnetic resonance cholangiopancreatography. N Engl J Med.

[53] Sugiyama M, Atomi Y, Hachiya J (1998). Magnetic resonance cholangiography using half-Fourier acquisition for diagnosing choledocholithiasis. Am J Gastroenterol.

[54] Liu CL, Lo CM, Chan JK, Poon RT, Lam CM, Fan ST (2001). Detection of choledocholithiasis by EUS in acute pancreatitis: a prospective evaluation in 100 consecutive patients. Gastrointest Endosc.

[55] Norton SA, Alderson D (2000). Endoscopic ultrasonography in the evaluation of idiopathic acute pancreatitis. Br J Surg.

[56] Brisinda G, Maria G, Ferrante A, Civello IM (1999). Evaluation of prognostic factors in patients with acute pancreatitis. Hepatogastroenterology.

[57] Matos C, Cappeliez O, Winant C, Coppens E, Deviére J, Metens T (2002). MR imaging of the pancreas: a pictorial tour. Radiographics.

[58] Formela LJ, Galloway SW, Kingsnorth AN (1995). Inflamatory mediators in acute pancreatitis. Br J Surg.

[59] Leese T, Shaw D, Holliday M (1988). Prognostic arkers in acute pancreatitis: can pancreatic necrosis be predicted?. Ann R Coll Surg Engl.

[60] Wilson C, Heads A, Shenkin A, Imrie CW (1989). C_reactive protein, antiproteases and complement factors as objective markers of severity of acute pancreatitis. Br J Surg.

[61] Gudgeon AM, Heath DI, Hurley P, Jehanli A, Patel G, Wilson C (1990). Trypsinogen activation peptides assay in the early prediction of severity of acute pancreatitis. Lancet.

[62] Uhl W, Büchler M, Malfertheiner P, Martini M, Beger HG (1991). PMN-eIastase in comparison with CRP, antiproteases, and LDH as indicators of necrosis in human acute pancreatitis. Pancreas.

[63] Gross V, Schölmerich J, Leser HG, Salm R, Lausen M, Rückauer K (1990). Granlllocyte elastase in assessment of severity of acute pancreatitis: comparison with acute-phase proteins C-reactive protein, alpha 1-antitrypsin, and protease inhibitor alpha2-macroglobulin. Dig Dis Sci.

[64] Manes G, Spada OA, Rabitti PG, Pacelli L, Iannaccone L, Uomo G (1997). Serum interleukin-6 in acute pancreatitis due to common bile duct stones. A reliable marker of necrosis. Recenti Prog Med.

[65] Neoptolemos JP, Carr-Locke DL, London NJ, Bailey IA, James D, Fossard DP (1988). Controlled trial of urgent endoscopic retrograde cholangiopancreatography and endoscopic sphincterotomy versus conservative treatment for acute pancreatitis due to gallstones. Lancet.

[66] Carroll BJ, Phillips EH (1993). The early treatment of acute biliary pancreatitis [letter; comment]. N Engl J Med.

[67] Fan ST, Lai EC, Mok FP, Lo CM, Zheng SS, Wong J (1993). Early treatment of acute biliary pancreatitis by endoscopic papillotomy. N Engl J Med.

[68] Stone HH, Fabian TC, Dunlop WE (1981). Gallstone pancreatitis: biliary tract pathology in relation to time of operation. Ann Surg.

[69] Kelly TR, Wagner DS (1988). Gallstone pancreatitis: a prospective randomized trial of the timing of surgery. Surgery.

[70] Fölsch UR, Nitsche R, Lüdtke R, Hilgers RA, Creutzfeldt W (1997). Early ERCP and papillotomy compared with conservative treatment for acute biliary pancreatitis. The German Study Group on Acute Biliary Pancreatitis N Engl J Med.

[71] Tarnasky PR, Cotton PB (1997). Early ERCP and papillotomy for acute biliary pancreatitis. N Engl J Med.

[72] Mergener K (1997). Early ERCP and papillotomy for acute biliary pancreatitis. N Engl J Med.

[73] Pasricha P (1997). Of opie, opossums, and others: emergent ERCP for gallstone pancreatitis. Gastroenterology.

[74] Tham TC, Lichtenstein DR, Vandervoort J, Wong RC, Brooks D, Van Dam J (1998). Role of endoscopic retrograde cholangiopancreatography for suspected choledocholithiasis in patients undergoing laparoscopic cholecystectomy. Gastrointest Endosc.

[75] Soetikno RM, Carr-Locke DL (1998). Endoscopic management of gallstone pancreatitis. Gastrointest Endosc Clin N Am.

[76] (2005). Working Party of the British Society of Gastroenterology; Association of Surgeons of Great Britain and Ireland; Pancreatic Society of Great Britain and Ireland; Association of Upper GI Surgeons of Great Britain and Ireland. UK guidelines for the management of acute pancreatitis. Gut.

[77] Sanjay P, Yeeting S, Whigham C, Judson H, Polignano FM, Tait IS (2008). Endoscopic sphincterotomy and interval cholecystectomy are reasonable alternatives to index cholecystectomy in severe acute gallstone pancreatitis. Surg Endosc.

[78] Williams EJ, Green J, Beckingham I, Parks R, Martin D, Lombard M (2008). British Society of Gastroenterology. Guidelines on the management of common bile duct stones (CBDS). Gut.

[79] Nowak A, Sowakowska-Dulawa E, Marek T, Rybicka J (1995). Final results of the prospective, randomized, controlled study on endoscopic sphincterotomy versus conventional management in acute biliary pancreatitis. Gastroenterology.

[80] Choudari CP, Lehman GA, Sherman S (1999). Pancreatitis and cystic fibrosis gene mutations. Gastroenterol Clin North Am.

[81] Freeman ML, Nelson DB, Sherman S, Haber GB, Herman ME, Dorsher PJ (1996). Complications of endoscopic biliary sphincterotomy. N Engl J Med.

[82] Freeman ML, DiSario JA, Nelson DB, Fennerty MB, Lee JG, Bjorkman DJ (2001). Risk factors for post-ERCP pancreatitis: a prospective, multicenter study. Gastrointest Endosc.

[83] Sherman S, Lehman GA (1997). Endoscopic therapy of pancreatic disease. The Gastroenterologist.

[84] Tarnasky PR, Hawes RH (1998). Endoscopic diagnosis and therapy of unexplained (idiopathic) acute pancreatitis. Gastrointest Endosc Clin N Am.

[85] Heider TR, Brown A, Grimm IS, Behrns KE (2006). Endoscopic sphincterotomy permits interval laparoscopic cholecystectomy in patients with moderately severe gallstone pancreatitis. J Gastrointest Surg.

[86] Uhl W, Müller CA, Krähenbühl L, Schmid SW, Schölzel S, Büchler MW (1999). Acute gallstone pancreatitis: timing of laparoscopic cholecystectomy in mild and severe disease. Surg Endosc.

[87] Chang L, Lo S, Stabile BE, Lewis RJ, Toosie K, de Virgilio C (2000). Preoperative versus postoperative endoscopic retrograde cholangiopancreatography in mild to moderate gallstone pancreatitis: a prospective randomized trial. Ann Surg.

[88] Schirmer B (2001). Timing of and indications for biliary tract surgery in acute necrotizing pancreatitis. J Gastrointest Surg.

[89] Barkun AN (2001). Early endoscopic management of acute gallstone pancreatitis -an evidence-based review. J Gastrointest Surg.

[90] Vitale GC (2007). Early Management of Acute Gallstone Pancreatitis. Ann Surg.

[91] Soper NJ (2001). Laparoscopic approach to the biliary tract in acute necrotizing pancreatitis. J Gastrointest Surg.

[92] Kim YJ, Kim MJ, Kim KW, Chung JB, Lee WJ, Kim JH (2005). Preoperative Evaluation of Common Bile Duct Stones in Patients with Gallstone Disease. AJR Am J Roentgenol.

